# Health care utilization and affordability among older people following China’s 2009 health reform -- evidence from CHARLS pilot study

**DOI:** 10.1186/s12939-019-0969-3

**Published:** 2019-05-03

**Authors:** Jiajia Li, Leiyu Shi, Hailun Liang, Chao Ma, Lingzhong Xu, Wen Qin

**Affiliations:** 1 School of Public Health, Shandong University, Jinan, 250012 China; 2 Key Laboratory of Health Economic and Policy Research, NHFPC, Shandong University, Jinan, 250012 China; 3 Bloomberg School of Public Health, Johns Hopkins University, Baltimore, 21205 USA; 4 School of Public health, Southeast University, Nanjing, China; 5 Infirmary of Shandong University, Jinan, 250012 China

**Keywords:** China’s health reform, Outpatient, Inpatient, Out-of-pocket, Pharmaceutical spending

## Abstract

**Background:**

In 2009, China unveiled an ambitious national health care reform program, with the goal of providing equitable and affordable basic health care for everyone. This study was intended to partially fill the knowledge gap in understanding of the demand-side impact on health care utilization and affordability among older people in Zhejiang and Gansu provinces of China.

**Methods:**

We used two waves of data from the pilot survey of CHARLS implemented in 2008 and 2012. Chi-square tests and t tests were performed to examine whether out-of-pocket (OOP) and pharmaceutical spending (PS), as a share of total health expenditures (THEs), have significantly changed following the health reform. Two-part model was employed to confirm these changes after controlling for confounding variables. All analyses were weighted and clustered the standard errors.

**Results:**

After controlling for confounding variables, older people in 2012 were 2.1 and 6.8% more likely to use outpatient and inpatient care than they did in 2008, respectively. Among those who have at least one outpatient visit, declines of OOP-to- THEs and PS-to-THEs percentage significantly reduced 0.998 (*p* < 0.1) and 2.324 (*p* < 0.01) from 2008 to 2012, respectively. However, conditional on having at least one inpatient stay, no significant reduction in terms of the OOP-to-THEs and even increase in terms of the PS-to-THEs percentage observed between 2008 and 2012. Compared to elderly people in Gansu, Zhejiang aged people had obviously better utilization, lighter inpatient OOP burden and lower inpatient PS proportion, but higher outpatient OOP burden and PS proportion.

**Conclusions:**

Although the OOP burden and PS portion had been reduced following the health reform, these impacts were still limited. Better results can be observed in outpatient care than in inpatient care, which provide a strong foundation for the next stage of reform.

## Introduction

Improving the utilization and affordability of health care is a high priority within countries and at the global level [1]. However, direct payments (out-of-pocket payments) in China account for a greater proportion of the sources of health-care financing than most of other Asian territories around the year of 2000 [2]. In an effort to solve this, the Chinese government has launched three social health insurance for different population groups with the aim of achieving universal coverage [3]. While the remarkable progress on health insurance coverage, existing evidences suggest limited, or even negative, impacts of health insurance schemes on relieving individuals financial burden of health care [3–7]. Under the marketization of medical services, the health insurance schemes may incentive both user and provider to provide more expensive care [5, 7].

To shift from market reliance to the reaffirmation of the government in financing and providing health care to the population [8], China unveiled an ambitious national health care reform program in April 2009. In the 2009–2011 phase of the reform, five main strategies were developed as the five major elements the reform, which included: expanding insurance coverage to more than 90% of the population, establishing a National Essential Medicines System (NEMS), improving the primary care delivery system, making public health services available and equal for all, and piloting public hospital reforms [8–11]. Detailed elaboration on the healthcare reform strategy has been described previously by Yip and Hsiao [9, 12]. Accordingly, a considerable amount, CN¥850 billion (US$125 billion), was invested in China’s health system from 2009 to 2011 [8, 9], which went into both the demand-side and the supply-side.

Ever since then, large-scale empirical studies have been conducted to investigate the effect of health care reform with mixed results in terms of the five main strategies: the effect of insurance expansion on individual co-payments [13]; the impact of NEMS on drug price and availability [14], on health service utilization and expenditures, on patients’ out-of-pocket burden [15, 16], and on institutional revenues and doctors’ incomes [17, 18]; the contribution of public hospital’s reform on patient and provider satisfaction [19], on prescribing patterns [20], on health resources’ allocation [21–23], and on total health expenditures (THEs) and out-of-pocket (OOP) payments [1]. However, the effects of the health care reform revealed by such indicators were focused on single-policy interventions on the supply-side or by patients’ records from the institutions and hospitals [1, 14, 17, 20–22, 24, 25], studies on the demand-side effect are limited.

Notwithstanding reform strategies and government investment into both the supply-side and the demand-side, the ultimate aim of the 2009 health reform was to improve the accessibility of basic health care, to reduce the individuals’ health burden, and to alleviate the longstanding problems and difficulties with high expenses in medical care (commonly known in Chinese as “Kan Bing Nan” and “Kan Bing Gui”) [26]. Citizens usually focus on what they can benefit from the reform rather than the reform itself [27]. The demand-side analysis would better reflect whether, and to what extent, people’s welfare gains have been promoted by the reform [15]. This study was therefore intended to partially fill the knowledge gap in understanding of the demand-side changes following the health reform.

In particular, we were interested in the middle-aged and elderly population’s feedback of the health reform. As one subgroup of the most vulnerable populations in China [3], older people were more sensitive to policy changes due to their higher risk of having chronic diseases and their lower socio-economic status. For the older people we concerned in this study, the reform may affect their access and affordability by the following efforts. First, the government increased the subsidization by 200 CNY per enrollee of New Cooperative Medical System (NCMS) and Urban Resident Basic Medical Insurance (URBMI) in 2011, to reduce individuals’ direct payments and improve the benefit of insurance programs. More than half of the enrolment subsidies came from the central government in western provinces like Gansu. Second, because of the historical phenomenon of provider-reliance on revenue from drug sales (known as “yi yao yang yi” in Chinese) [16], the government introduced NEMS to deal with the dramatically high drug expenses [15, 28].The public primary health-care institutions can only prescribe drugs from the National Essential Medicine List with zero mark-up policy and higher insurance reimbursement. Third, to provide better prevention and care for vulnerable populations like older people and residents with non-communicable diseases (NCDs), the government increased investment to primary health-care institutions for them to deliver a free package of public health services for the population in their catchment areas.

Using demand-side data from the pilot survey of the China Health and Retirement Longitudinal Study (CHARLS), this study evaluated the health care utilization and affordability placed on the elderlies following the 2009 health reform. Moreover, considering all of the other four components of the health reform have linkage with medicines, the share of pharmaceutical spending was also taken into account to reflect the comprehensive impact of the reform. Three main hypotheses were evaluated: 1) If the probability of using health services has been improved? 2) If older people relied less heavily on OOP when they use health services? 3) If the pharmaceutical spending (PS) as a share of THEs has been reduced effectively?

## Materials and methods

### Data

We used two waves of data from the pilot survey of CHARLS implemented in 2008 and 2012, which were collected just before and after the national health reform initialization in 2009. CHARLS, as a national survey, targeted the population aged 45 and above. The detail of the pilot data has been described in previous studies [3, 29, 30]. The pilot survey was conducted in two distinct provinces: Gansu, a poor agricultural province on the Northwest Inland, and Zhejiang, a rich industrialized province on the East Coast. The survey included 2685 interviewees living in 95 communities/villages in 32 counties/districts. We excluded samples with missing values in our analysis, leaving 2248 observations in 2008 and 2121 observations in 2012.The Institutional Review Board of Peking University granted ethical consent.

### Measures/variables

To estimate the impact of the health reform in 2009 from a demand-side view, we investigated whether health care utilization had been improved, and whether the health burden and cost of drugs had been reduced between 2008 and 2012. The outpatient and inpatient utilization was measured based on the following questions: (1) Had there been an outpatient visit during the last month (coded as binary)? (2) Had there been an inpatient stay during the last year (coded as binary)? (3) How many times did you visit medical facilities during the last month (totaling outpatient visits of all type medical facilities)? (4) How many times have you received inpatient care during the past year (times)?

Since the nature of longitudinal data, samples in 2012 got older, faced worse health and higher prices than in 2008. Therefore, we use the OOP share of THEs rather than OOP expenditure to measure the burden of health expenditures placed on individuals [31]. We used OOP-to-THEs percentage of the most recent outpatient visit and OOP-to-THEs percentage of the most recent inpatient stay to measure the outpatient burden and inpatient burden, respectively. We chose the variables of the most recent outpatient and inpatient visits, rather than those of outpatients during the last month and inpatients during the last year, because the sample size of the latter two groups was too small.

Similarly, to measure the spending on medicines, we selected two variables: (1) PS-to-THEs percentage of the most recent outpatient visit; (2) PS-to-THEs percentage of the most recent inpatient stay.

The key independent variable to evaluate the changes following the 2009 health reform was the time-related variable: before reform (2008) and after reform (2012).Besides the time dummy variable, we used observable individual characteristics to control the time-varying characteristics and to adjust the sample heterogeneity between the 2 years. According to the Andersen/Aday Health Behaviour Model and previous studies [32–34], usage of health services was determined by need factors, enabling factors, and predisposing factors: (1) health need characteristics, including activities of daily living (without ADL difficulty / with at least one ADL difficulty), self-reported health status (categorized as excellent/good health, fair health, and poor/very poor health); (2) predisposing variables, including age (years), gender (male/female), marital status (married/others); (3) enabling factors, including education level (classified as illiterate, below primary but can read/write, primary school, junior high school and above), household expenditure per capita (RMB in 2012 value), province (Zhejiang/ Gansu),and some, types of medical insurance (classified into none, NCMS, URBMI, UEBMI (Urban Employee Basic Medical Insurance), IBMI (Integrated Urban-rural Basic Medical Insurance), and others (including commercial medical insurance, government free medical insurance)), method of reimbursement (get reimbursement later /get reimbursement immediately). Those policy-related and health care accessibility variables which included in the enabling factors could also make sense to capture the supply-side changes in the health reform. Table 3 in Appendix provides a list and a summary of descriptive statistics for the independent variables used in this study.

### Statistical analysis

Descriptive statistics for health care utilization, health burden, and spending of drugs before and after the 2009 health reform reported estimates as means for continuous variables and proportions for categorical variables. To examine whether there were statistically significant differences between 2008 and 2012, we conducted Chi-square tests for dichotomous variables and T-tests for continuous variables and reported their *p*-values. In addition, since the pilot survey of CHARLS focused on two very different provinces of China - Zhejiang and Gansu, we analyzed the changes following the health reform separately.

Although CHARLS pilot data is a longitudinal data, the samples who used health services did not necessarily overlap in 2008 and 2012, due to the randomness of illness. Among the 1989 panel samples, only 107 used outpatient care in both years, and only 23 were hospitalized in both years. Therefore, we used pooled regression instead of panel regression by adopting two-part model, to investigate the demand-side impact of health reform in multivariate analyses adjusted for confounding variables. The first part is a logistic regression that predicts the probability of health care utilization, and the second part is a generalized linear model (GLM) model for the burdens of health care and PS proportion conditional on at least one visit to a service provider. We used the individual sample weights to produce population representative estimates and clustered the standard errors at the individual level in the pooled regression. Adjusted odds ratios and coefficients with their *p*-values were reported. Data analyses were conducted by using survey commands with STATA 14.0.

## Results

Table 1 displays theutilization, OOP burden and PS proportion of outpatient services and hospital services by province, as well as, before and after reform. When compared with older people in 2008, those in 2012 were more likely to use outpatient services and hospital services in both provinces. Relative to 2008, the percentage of outpatient visits in 2012 significantly increased from 17.93 to 22.43% (*p* < 0.05) in Gansu, and increased from 21.72 to 23.86% (*P* = 0.500) in Zhejiang. Similarly, the proportion of hospitalization sharply increased from 8.40% in 2008 to 15.85% in 2012 in Zhejiang, with an increase rate of 88.69%, and the significant level at *p* < 0.01; while the increase rate of hospitalization in Gansu was only 8.90%, from 6.63% in 2008 to 7.22% in 2012 (*p* = 0.104). Apart from proportions, the frequency of hospitalization increased from 2008 to 2012 in both Gansu and Zhejiang. The frequency of outpatient visits increased for the aged in Gansu but decreased for their counterparts in Zhejiang between 2008 and 2012.

**Table 1:** Healthcare utilization, Health burden, and PS proportion before and after 2009 health reform

Variables	All	2008	2012	P
*Panel A: Zhejiang*				
Outpatient				
% of outpatient visit, %	22.72	21.72	23.86	0.500
Frequency of outpatient visits, mean(SD)	2.46 (0.12)	2.64 (0.19)	2.26 (0.16)	0.133
OOP-to-THEs %, mean(SD)	86.95 (1.44)	90.74 (1.97)	82.51 (2.56)	0.004
PS-to-THEs %, mean(SD)	81.89 (1.89)	86.86 (2.03)	76.30 (2.84)	0.000
Inpatient				
% of being hospitalized, %	11.86	8.40	15.85	0.000
Frequency of hospitalization, mean(SD)	1.34 (0.05)	1.27 (0.06)	1.38 (0.07)	0.306
OOP-to-THEs%, mean(SD)	61.02 (2.51)	61.24 (3.97)	60.88 (3.17)	0.552
PS-to-THEs%, mean(SD)	55.68 (3.66)	54.19 (6.44)	56.74 (3.99)	0.910
*Panel B: Gansu*				
Outpatient				
% of outpatient visit, %	20.11	17.93	22.43	0.011
Frequency of outpatient visits, mean(SD)	2.16 (0.11)	2.09 (0.16)	2.22 (0.14)	0.537
OOP-to-THEs%, mean(SD)	74.76 (5.00)	77.89 (5.66)	71.83 (5.11)	0.226
PS-to-THEs%, mean(SD)	69.25 (2.52)	79.25 (2.93)	61.20 (3.83)	0.004
Inpatient				
% of being hospitalized, %	6.91	6.63	7.22	0.104
Frequency of hospitalization, mean(SD)	1.24 (0.08)	1.13 (0.40)	1.33 (0.13)	0.203
OOP-to-THEs%, mean(SD)	61.41 (4.77)	61.78 (6.80)	60.99 (5.05)	0.624
PS-to-THEs%, mean(SD)	41.68 (4.48)	41.51 (4.28)	41.88 (7.17)	0.806

The proportions reported in the table are weighted proportions. The means reported in the table are weighted means, and robust standard errors (Ses) are in parentheses, all stratified by county level and clustered at PSU. χ^2^ tests for dichotomous variables and t-tests for continuous variables

Discrepancies were found in the OOP share of THEs between the 2 years. In Zhejiang, the OOP-to-THEs percentage of the most recent outpatient visit dropped remarkably from 90.74% in 2008 to 82.51% in 2012 (*p* < 0.01), whereas the percentage of the most recent hospitalization measured little changes from 2008 to 2012. (61.24% vs. 60.88%, *p* = 0.552). Similarly, percentage of OOP-to-THEs in 2012 was lower when compared with that in 2008 in Gansu (outpatient: 77.89% vs. 71.83%, *p* = 0.226; inpatient: 61.78% vs. 60.99%, *p* = 0.624).

Comparably, statistically significant decreases in the PS-to-THEs percentage of the most recent outpatient visit were observed between 2008 and 2012 in both provinces (Zhejiang: 86.86% vs. 73.30%, *p* < 0.01; Gansu: 79.25% vs.61.20%, *p* < 0.01). However, the PS-to-THEs percentage of the most recent inpatient stay insignificantly increased in both province.

Table 2 presents the results of the two-part model for healthcare utilization, health burden and cost of drugs of outpatient services and hospital services. After controlling for confounding variables, individuals in 2012 were more likely to use healthcare than they did in 2008. The improvement on healthcare use was stronger for the inpatient stay than the outpatient visit: older population in 2012 were 2.1% more likely to use outpatient services (OR = 1.021) and 6.8% more likely to use hospital services (OR = 1.068) than they did in 2008, respectively.

**Table 2:** Two-part model: utilization and affordability

VARIABLES	Selection equation: utilization > 0	Outpatient			Inpatient	
		Outcome equation: conditional OOP%	Outcome equation: conditional PS%	Selection equation: utilization > 0	Outcome equation: conditional OOP%	Outcome equation: conditional PS%
Health Reform:2012	1.021	−0.998*	−2.324***	1.068	−0.217	1.539
	(0.025)	(0.583)	(0.831)	(0.0458)	(0.908)	(0.994)
Province: Zhejiang	1.383**	7.288**	7.817**	1.583***	−3.427	−1.197
	(0.188)	(3.058)	(3.432)	(0.235)	(4.435)	(6.736)
Age	1.007	−0.00543	0.405**	1.034***	− 0.382**	0.181
	(0.006)	(0.134)	(0.188)	(0.00774)	(0.189)	(0.326)
Gender: Female	1.697***	−0.288	5.164*	0.821	−5.350*	2.439
	(0.214)	(1.791)	(2.769)	(0.121)	(3.209)	(5.811)
Marital status: Others	1.027	3.227	−0.597	0.775	9.122**	11.83**
	(0.128)	(2.702)	(4.563)	(0.141)	(3.724)	(5.803)
Education level:	1.644***	−0.363	1.728	0.954	−3.507	0.349
Can read/writing	(0.253)	(2.718)	(4.277)	(0.202)	(4.254)	(6.402)
Education level:	1.538**	−0.655	0.377	0.981	−8.724*	−8.845
Primary school	(0.275)	(2.501)	(4.424)	(0.163)	(4.557)	(8.851)
Education level:	1.282*	−1.596	−1.835	0.966	−10.43**	6.469
Junior high and above	(0.174)	(2.572)	(4.076)	(0.190)	(4.207)	(6.483)
Expenditure	1.117**	1.291*	−1.778*	1.142*	1.868	−2.593
(RMB in 2012 value)	(0.0503)	(0.710)	(0.914)	(0.0820)	(1.697)	(3.091)
ADL:	1.291*	−0.968	−0.736	1.583**	8.296**	10.52*
With any ADL	(0.181)	(2.445)	(3.056)	(0.298)	(4.114)	(6.147)
Self-reported health:	2.147***	−2.540	−4.277	1.691***	−7.541	−14.13**
Fair health	(0.237)	(2.916)	(4.178)	(0.325)	(5.629)	(6.455)
Self-reported health:	3.952***	−1.679	−6.893	3.734***	−10.34*	− 12.59*
Poor/very poor health	(0.572)	(2.676)	(4.490)	(0.637)	(5.585)	(6.582)
Types of insurance:	0.912	−4.532**	8.784*	1.321	−24.31***	0.721
NCMS	(0.177)	(1.782)	(5.114)	(0.489)	(3.039)	(4.061)
Types of insurance:	1.275	−16.33**	3.388	1.831	−34.41***	−5.620
URBMI	(0.351)	(6.671)	(8.567)	(0.862)	(2.859)	(15.98)
Types of insurance:	1.651	− 39.65***	12.10*	1.767	−48.90***	−2.760
UEBMI	(0.507)	(8.006)	(6.987)	(0.726)	(7.024)	(6.012)
Types of insurance:	1.262	−14.33**	8.904	1.312	−51.00***	−1.348
Others	(0.324)	(6.590)	(7.966)	(0.621)	(8.751)	(7.997)
Types of insurance:	1.469	0.975	20.08**	1.049	−17.65***	51.99***
IBMI	(0.450)	(2.371)	(8.945)	(0.550)	(3.990)	(7.373)
Get reimbursement	1.440***	−4.581*	−2.543	0.991	−0.590	−13.78**
Immediately	(0.152)	(2.411)	(3.524)	(0.167)	(4.137)	(6.080)
Constant	678.1***	2092*	4732***	−96.12	550.2	− 3017
	(192.0)	(1171)	(1672)	(95.55)	(1817)	(1989)

Robust standard errors are reported in parenthesis.

***p < 0.01, ** *p* < 0.05, * *p* < 0.1

Conditional on having any outpatient visit, health reform reduced the percentage of OOP-to- THEs by 0.998. Similarly, a decline of PS-to- THEs percentage significantly reduced 2.324 from 2008 to 2012. However, among those who had at least one outpatient or inpatient visit, there was no significant difference in terms of the OOP-to-THEs percentage between 2008 and 2012. The PS-to- THEs of the most recently inpatient stay in 2012 was even higher than that in 2008.

After controlling for confounding variables, older people in Zhejiang were respectively 38.3 and 58.3% more likely to have outpatient visit and inpatient care, when compared with older people in Gansu. However, Zhejiang aged people had heavier outpatient OOP burden and higher PS proportion than their Gansu counterparts. Among those who had at least one inpatient service utilization, the inpatient OOP burden and PS proportion was lower for older people in Zhejiang than that for those in Gansu.

In terms of other covariates, we found that older people with higher expenditure significantly had better utilization of both outpatient and inpatient services, and conditionally had higher OOP proportion and lower PS proportion as share of THEs. Compared with those without insurance, people with any type of insurance were more likely to have outpatient visit and inpatient care, except the negative impact of NCMS on outpatient care. Conditional on having at least one outpatient visit, NCMS, URBMI, UEBMI, and Others type of insurance significantly reduced the percentage of outpatient OOP-to-THEs by 4.532, 16.33, 39.65, and 14.33, respectively, whereas all kinds of insurance increase the PS-to-THEs percentage. The reductions of insurance on inpatient OOP-to-THEs were more drastic than those on outpatient. In addition, people with higher education tended to face lower OOP burden in both outpatient service and hospital service.

## Discussion

From the demand-side perspective, the present study reveals a significant improvement in healthcare utilization for both Zhejiang and Gansu older people following the implementation of health reform. However, the improvement become insignificant after controlling for confounding factors, which was consistent with the study by Meng et al. [11]. The longitudinal data structure of CHARLS provides the most straightforward explanation: the samples aged 4 years from 2008 to 2012. Additionally, the proportions of both outpatient and inpatient care use in 2012 were higher than previous studies using adult data [11], which demonstrated that the elderly population were more sensitive to policy changes than the population at large. The increase on outpatient care utilization was more obviously in Gansu than in Zhejiang, while the changes on hospital services use was greater in Zhejiang than in Gansu.

Consistent with previous studies, there was a significant reduction in the OOP-to-THEs ratio after the 2009 health reform [25, 31, 35], indicating the positive effect of China’s health reform, especially on strategies that increased government financial investment in health insurance plans’ improvement. Conditional upon use, the OOP-to-THEs percentage of the outpatient care for both Zhejiang and Gansu older population showed apparently decreased, with no obvious changes to the inpatient OOP-to-THEs percentage. However, the fact is that the health insurance mainly covered inpatient costs with ever-increasing reimbursement rates [9, 11], outpatient care is not yet covered in Gansu and just beginning to be covered in Zhejiang [36]. As previous empirical evidence suggested, expanding insurance or reducing patient co-payment would be complicated by a strategic supply-side response [16], which indicated that actions targeting both providers and consumers were equally important in the reform. Additionally, although older people’s OOP burden were diminished after the reform, the direct payments of outpatient visits and inpatient stays were still account for 79 and 61% of THEs, respectively, much higher than average proportion in China and most of other Asian countries [2]. Large public investments did not seem to have offered strong financial protection for elderly people. Therefore, subsidizing mechanisms should be given more attention to protect against the older people’s health burden.

Similarly, the share of PS on outpatient visits declined significantly from 2008 to 2012. The implication of NEMS in the 2009 health reform may have contributed to this decrease. Since the mark-up rate of public hospital is fixed by regulation, a large proportion of health providers’ revenue came from pharmaceutical sales (known as “yi yao yang yi” in Chinese) [16]. Although the first National Essential Medicine List (NEML) had been developed in 1981, whereas the effect was poor since lack of appropriate supporting policies and mechanisms [37]. Therefore, the most important policy of NEMS in 2009 reform is to implement a zero mark-up policy in government-owned primary care organizations (known as urban community health care centers and rural township hospitals), with the explicit target of eliminating the provider-reliance on pharmaceutical profits. Previous studies proved early policy success on reducing the number of medicines and costs-per-prescription in primary healthcare institutions [20, 38]. However, the zero mark-up policy on hospitals has only been piloted in recent years and would be spread in second phase of the reform. As a result, our research observes a more noticeable reduction of PS proportion on outpatient visits, but an increase on inpatient stays. Moreover, although the PS proportion in outpatient visits were diminished following the reform, spending on medicines still accounted for 66.63% of outpatient expenditures and 49.60% of inpatient expenditures in 2012, while the proportion in OECD countries only averaging around 17% [39]. Since more than half of the health care personnel and health care expenditures were distributed in public hospitals, it seems more important now than ever before to spreads the reform into public hospitals, and better outcomes can be expected then.

We also found that the interprovincial difference in outpatient utilization decreased following the health reform. Health reform strategies of improving primary care and promoting public health services might have played a positive role in the narrowing of the gap [29, 40]. There are only 377 hospitals and 9793 primary health care facilities throughout Gansu province in 2008, compared with 635 hospitals and 14,028 primary health care facilities in Zhejiang [41]. The ‘inconvenient traffic’, ‘being poor’, and ‘no available treatment’ were more important reasons for poor utilization in Gansu than in Zhejiang [36]. Fortunately, in 2012 following the 2009–2011 phase of the health reform, the number of primary health care facilities in Gansu dramatically increased to 25,931, which were comparable to Zhejiang with the number of 28,939. However, large provincial disparities still exist in high-quality health resources even after the reform: the number of hospitals in Gansu is only half that of Zhejiang (403 vs. 782) [42]. This is consistent with revelation in this study: although samples in Zhejiang have obviously better use of health service than their Gansu counterparts, they faced higher OOP burden and PS proportion. Accordingly, the government should strengthen the quality of health service in undeveloped regions while encouraging the use of primary care in developed regions.

## Limitations

The results from the present study should be interpreted with caution, as there are some limitations. First, as researchers has suggested, there are enormous challenges in assessing the impact of the reforms because the changes are applied across the entire country [8], we can only study the correlation rather than the causal effect of the health reform. Second, although panel regression would perform better in addressing the unobserved individual effects, due to the nature of our dependent variables, we used pooled regression to capture the changes following the health reform. Third, to reflect the changes following the reform as objectively as possible, we used the OOP share of THEs rather than OOP expenditure to measure the OOP burden, but we can only demonstrate the relative changes rather than absolute changes before and after the reform. Fourth, As the THEs, OOP and PS were collected through respondents’ retrospective investigation, which may lead to measurement bias.

## Conclusions

Despite these limitations, this study provides references for health reform in China and other developing countries from the demand-side perspective. Also, we particularly focus on older people, which was one of the subgroup of vulnerable populations and lack of adequate attention. The present study showed that among the Chinese population aged 45 and older in Zhejiang and Gansu provinces, although the utilization and affordability of healthcare improved following China’s 2009 health reform, the impacts were limited. The older people in both province still faced heavily OOP burden and high pharmaceutical spending proportion. Although with lighter health burden, the older people in Gansu were underutilizing health care when compared to their counterparts in Zhejiang. More worrisome is that we find even higher share of PS for a hospitalization from 2008 to 2012, which indicate that the reform effect on hospital service is still far from ideal. The first phase from 2009 to 2011 is a key period for the reform, which has an important reference for the following reform. As one of the most vulnerable populations and the major user of health services, we recommend that different efforts should be made to improve the well-being of older people in different regions. First, for regions like Gansu where medical resources are scarce, we should promote accessibility of health care through strengthening the medical supply and other means including eHealth. Second, for resourced regions like Zhejiang, we should strengthen primary care services and deepen public hospital reform to lead to hierarchical medical use. Third, social health insurance should offer better coverage of NCDs, which is the almost the most important source of health burden for the elderly, to provide them stronger financial protection.

## Appendix

**Table 3:** Respondents characteristics

Variables	All	2008	2012	P
Age	60.89 (0.15)	59.86 (0.35)	63.18 (0.31)	0.000
Female	49.85	49.92	49.76	0.536
Single	17.60	17.54	17.67	0.794
Education level				0.751
Illiterate (reference)	40.16	40.40	39.90	
Below primary but can read/write	20.71	20.18	21.29	
Primary school	17.64	17.33	17.99	
Junior high school and above	21.48	22.08	20.83	
Log (Household expenditure per capita) (RMB in 2012 value)	8.62 (0.06)	8.65 (0.06)	8.57 (0.07)	0.316
With at least one ADL difficulty	11.86	9.56	14.37	0.000
Self-reported health status				0.054
Excellent/good health	32.36	33.40	31.22	
Fair health	45.85	43.55	48.36	
Poor/very poor health	21.80	23.05	20.43	
Types of medical insurance				0.000
None (reference)	6.49	8.03	4.81	
NCMS	72.12	74.86	69.11	
URBMI	4.22	3.24	5.29	
UEBMI	12.25	11.37	13.22	
IBMI	2.90	0	3.34	
Others	2.02	2.50	4.23	
Get reimbursement immediately	43.12	31.04	56.34	0.000
observations	4369	2248	2121	

The proportions reported in the table are weighted proportions. The means reported in the table are weighted means, and robust standard errors (Ses) are in parentheses, all stratified by county level and clustered at PSU. χ^2^ tests for dichotomous variables and t-tests for continuous variables

## Abbreviations

CHARLS: China Health and Retirement Longitudinal Study
GLM: Generalized linear model
IBMI: Integrated urban-rural basic medical insurance
NCDs: Non-communicable diseasesADL, activities of daily living
NCMS: New cooperative medical system in rural
NEMS: National Essential Medicines System
OOP: Out-of-pocket
PS: Pharmaceutical spending
THEs: total health expenditures
UEBMI: Urban employee basic medical insurance
URBMI: Urban resident basic medical insurance

## References

[1] Jian WY, Lu M, Chan KY, Poon AN, Han W, Hu M, Yip W (2015). Payment reform pilot in Beijing hospitals reduced expenditures and out-of-pocket payments per admission. Health Aff.

[2] O'Donnell O, Van Doorslaer E, Rannan-Eliya RP, Somanathan A, Adhikari SR, Akkazieva B, Harbianto D, Garg CC, Hanvoravongchai P, Herrin AN (2008). Who pays for health care in Asia. J Health Econ.

[3] Li X, Zhang W (2013). The impacts of health insurance on health care utilization among the older people in China. Soc Sci Med.

[4] Lei XY, Lin WC (2009). The new cooperative medical scheme in rural China: does more coverage mean more service and better health?. Health Econ.

[5] Wagstaff A, Lindelow M (2008). Can insurance increase financial risk? The curious case of health insurance in China. J Health Econ.

[6] Atella V, Brugiavini A, Pace N (2015). The health care system reform in China: effects on out-of-pocket expenses and saving. China Econ Rev.

[7] Hou ZY, Van de Poel E, Van Doorslaer E, Yu BR, Meng QY (2014). Effects of NCMS on access to care and financial protection in China. Health Econ.

[8] Powell-Jackson T, Yip WCM, Han W (2015). Realigning demand and supply side INCENTIVES to improve primary health care seeking in rural China. Health Econ.

[9] Yip WC-M, Hsiao WC, Chen W, Hu S, Ma J, Maynard A (2012). Early appraisal of China's huge and complex health-care reforms. Lancet.

[10] Yang JQ, Hong YM, Ma SG (2016). Impact of the new health care reform on hospital expenditure in China: a case study from a pilot city. China Econ Rev.

[11] Meng Q, Xu L, Zhang YG, Qian JC, Cai M, Xin Y, Gao J, Xu K, Boerma JT, Barber SL (2012). Trends in access to health services and financial protection in China between 2003 and 2011: a cross-sectional study. Lancet.

[12] Yip W, Hsiao W (2009). China's health care reform: a tentative assessment. China Econ Rev.

[13] Dong H, Duan S, Bogg L, Wu Y, You H, Chen J, Ye X, Seccombe K, Yu H (2016). The impact of expanded health system reform on governmental contributions and individual copayments in the new Chinese rural cooperative medical system. Int J Health Plann Manag.

[14] Fang Y, Wagner AK, Yang S, Jiang M, Zhang F, Ross-Degnan D (2013). Access to affordable medicines after health reform: evidence from two cross-sectional surveys in Shaanxi Province, western China. Lancet Glob Health.

[15] Ding L, Wu J (2017). The impact of China's National Essential Medicine Policy and its implications for urban outpatients: a multivariate difference-in-differences study. Value Health.

[16] Chen BK, Yang YT, Eggleston K (2017). Patient copayments, provider Incentives, and income effects: theory and evidence from the essential medications list under China's 2009 healthcare reform. World Med Health Policy.

[17] Guo Z, Guan X, Shi L (2017). The impacts of implementation of National Essential Medicines Policies on primary healthcare institutions: a cross-sectional study in China. BMC Health Serv Res.

[18] Xu S, Bian C, Wang H, Li N, Wu J, Li P, Lu H. Evaluation of the implementation outcomes of the essential medicines system in Anhui county-level public hospitals: a before-and-after study. BMC Health Serv Res. 2015;15:403. 10.1186/s12913-015-1073-z PMC4579796 26394615

[19] Cheng T-M (2013). A pilot project using evidence-based clinical pathways and payment reform in China's rural hospitals shows early success. Health Aff.

[20] Ding Ding, Pan Qingxia, Shan Linghan, Liu Chaojie, Gao Lijun, Hao Yanhua, Song Jian, Ning Ning, Cui Yu, Li Ye, Qi Xinye, Liang Chao, Wu Qunhong, Liu Guoxiang (2016). Prescribing Patterns in Outpatient Clinics of Township Hospitals in China: A Comparative Study before and after the 2009 Health System Reform. International Journal of Environmental Research and Public Health.

[21] Fang P, Hu R, Han Q (2017). Effects of healthcare reform on health resource allocation and service utilization in 1110 Chinese county hospitals: data from 2006 to 2012. Int J Health Plann Manag.

[22] Wong HT, Guo YQ, Chiu MYL, Chen S, Zhao Y (2016). Spatial illustration of health-care workforce accessibility index in China: how far has our 2009 health-care reform brought us?. Aust J Rural Health.

[23] Yang Q, Dong H (2014). Have health human resources become more equal between rural and urban areas after the new reform?. Int J Health Policy Manag.

[24] Liu Q, Tian X, Tian J, Zhang X (2014). Evaluation of the effects of comprehensive reform on primary healthcare institutions in Anhui Province. BMC Health Serv Res.

[25] Wang S, Liu L, Li L, Liu J (2014). Comparison of Chinese inpatients with different types of medical insurance before and after the 2009 healthcare reform. BMC Health Serv Res.

[26] Li XL, Miao YQ, Chen WJ (2015). China's three-year health reform program and equity in sanitation improvement: a panel analysis. BMC Public Health.

[27] Qin X, Luo H, Feng J, Li Y, Wei B, Feng Q (2017). Equity in health financing of Guangxi after China's universal health coverage: evidence based on health expenditure comparison in rural Guangxi Zhuang autonomous region from 2009 to 2013. Int J Equity Health.

[28] Chen Yixi, Hu Shanlian, Dong Peng, Kornfeld Åsa, Jaros Patrycja, Yan Jing, Ma Fangfang, Toumi Mondher (2016). Drug pricing reform in China: analysis of piloted approaches and potential impact of the reform. Journal of Market Access & Health Policy.

[29] Hou Z, Meng Q, Zhang Y (2016). Hypertension prevalence, awareness, treatment, and control following China's healthcare reform. Am J Hypertens.

[30] Feng XL, Pang MF, Beard J (2014). Health system strengthening and hypertension awareness, treatment and control: data from the China health and retirement longitudinal study. Bull World Health Organ.

[31] Zhang LF, Liu N (2014). Health reform and out-of-pocket payments: lessons from China. Health Policy Plan.

[32] Aday LA, Andersen R (1974). A framework for the study of access to medical care. Health Serv Res.

[33] Aday LA, Andersen RM (1981). Equity of access to medical-care - a conceptual and empirical overview. Med Care.

[34] Aday LA, Andersen RM (1984). The national profile of access to medical-care - where do we stand. Am J Public Health.

[35] Deng F, Lv JH, Wang HL, Gao JM, Zhou ZL (2017). Expanding public health in China: an empirical analysis of healthcare inputs and outputs. Public Health.

[36] Mu R (2014). Regional disparities in self-reported health: evidence from CHINESE older adults. Health Econ.

[37] Chen W, Tang S, Sun J, Ross-Degnan D, Wagner AK (2010). Availability and use of essential medicines in China: manufacturing, supply, and prescribing in Shandong and Gansu provinces. BMC Health Serv Res.

[38] Song Y, Bian Y, Petzold M, Li L, Yin A (2014). Effects of the National Essential Medicine System in reducing drug prices: an empirical study in four Chinese provinces. J Pharm Policy Pract.

[39] Barber SL, Huang B, Santoso B, Laing R, Paris V, Wu C (2013). The reform of the essential medicines system in China: a comprehensive approach to universal coverage. J Glob Health.

[40] Zhang L, Cheng G, Song S, Yuan B, Zhu W, He L, Ma X, Meng Q (2017). Efficiency performance of China's health care delivery system. Int J Health Plann Manag.

[41] Ministry of Health of China. China health statistics yearbook (2009). Beijing: Beijing Union Medical University Press; 2009.

[42] Ministry of Health of China. China health statistics yearbook (2013). Beijing: Beijing Union Medical University Press; 2013.

